# Examination of the neural basis of psychotic-like experiences in adolescence during processing of emotional faces

**DOI:** 10.1038/s41598-020-62026-7

**Published:** 2020-03-20

**Authors:** Evangelos Papanastasiou, Elias Mouchlianitis, Dan W. Joyce, Philip McGuire, Celia Boussebaa, Tobias Banaschewski, Arun L. W. Bokde, Christian Büchel, Erin Quinlan, Sylvane Desrivières, Herta Flor, Antoine Grigis, Hugh Garavan, Philip Spechler, Penny Gowland, Andreas Heinz, Bernd Ittermann, Marie-Laure Paillère Martinot, Eric Artiges, Frauke Nees, Dimitri Papadopoulos Orfanos, Tomáš Paus, Luise Poustka, Sabina Millenet, Juliane H. Fröhner, Michael N. Smolka, Henrik Walter, Robert Whelan, Gunter Schumann, Sukhwinder S. Shergill

**Affiliations:** 1 Cognition Schizophrenia and Imaging Laboratory, Department of Psychosis Studies, Institute of Psychiatry Psychology and Neuroscience, King’s College London, De Crespigny Park, Denmark Hill, London, SE5 8AF United Kingdom; 2 Cognition Schizophrenia and Imaging Laboratory, Department of Psychosis Studies, Institute of Psychiatry Psychology and Neuroscience, King’s College London, London, United Kingdom; 3 Department of Psychosis Studies, Institute of Psychiatry Psychology and Neuroscience, King’s College London, London, United Kingdom; 4 Department of Child and Adolescent Psychiatry and Psychotherapy, Central Institute of Mental Health, Medical Faculty Mannheim, Heidelberg University, Mannheim, Germany; 5 Discipline of Psychiatry, School of Medicine and Trinity College Institute of Neuroscience, Trinity College Dublin, Dublin, Ireland; 6 Department of Systems Neuroscience, University Medical Centre Hamburg-Eppendorf, Hamburg, Germany; 7 Centre for Population Neuroscience and Precision Medicine, Institute of Psychiatry Psychology and Neuroscience, King’s College London, London, United Kingdom; 8 Medical Research Council, Social Genetic and Developmental Psychiatry Centre, Institute of Psychiatry Psychology and Neuroscience, King’s College London, London, United Kingdom; 9 Department of Cognitive and Clinical Neuroscience, Central Institute of Mental Health, Medical Faculty Mannheim, Heidelberg University, Mannheim, Germany; 10 Department of Psychology, School of Social Sciences, University of Mannheim, Mannheim, Germany; 11 NeuroSpin, CEA, Université Paris-Saclay, Gif-sur-Yvette, France; 12 Department of Psychiatry, University of Vermont, Burlington, Vermont, USA; 13 Department of Psychology, University of Vermont, Burlington, Vermont, USA; 14 Sir Peter Mansfield Imaging Centre School of Physics and Astronomy, University of Nottingham, Nottingham, United Kingdom; 15 Department of Psychiatry and Psychotherapy, Campus Charité Mitte, Charité, Universitätsmedizin Berlin, Berlin, Germany; 16 Physikalisch-Technische Bundesanstalt, Berlin, Germany; 17 Institut National de la Santé et de la Recherche Médicale, Neuroimaging & Psychiatry, University Paris Sud – Paris-Saclay, University Paris Descartes, Paris, France; 18 Department of Adolescent Psychopathology and Medicine, Maison de Solenn, Cochin Hospital, Paris, France; 19 Institut National de la Santé et de la Recherche Médicale, Neuroimaging & Psychiatry, DIGITEO Labs, University Paris Saclay, Gif-sur-Yvette, France; 20 Psychiatry Department, Orsay Hospital, Orsay, France; 21 Bloorview Research Institute, Holland Bloorview Kids Rehabilitation Hospital and Departments of Psychology and Psychiatry, University of Toronto, Toronto, Ontario Canada; 22 Department of Child and Adolescent Psychiatry and Psychotherapy, University Medical Centre Göttingen, Göttingen, Germany; 23 Clinic for Child and Adolescent Psychiatry, Medical University of Vienna, Vienna, Austria; 24 Department of Psychiatry and Neuroimaging Centre, Technische Universität Dresden, Dresden, Germany; 25 School of Psychology and Global Brain Health Institute, Trinity College Dublin, Dublin, Ireland

**Keywords:** Cognitive neuroscience, Human behaviour, Diagnostic markers

## Abstract

Contemporary theories propose that dysregulation of emotional perception is involved in the aetiology of psychosis. 298 healthy adolescents were assessed at age 14- and 19-years using fMRI while performing a facial emotion task. Psychotic-like experiences (PLEs) were assessed with the CAPE-42 questionnaire at age 19. The high PLEs group at age 19 years exhibited an enhanced response in right insular cortex and decreased response in right prefrontal, right parahippocampal and left striatal regions; also, a gradient of decreasing response to emotional faces with age, from 14 to 19 years, in the right parahippocampal region and left insular cortical area. The right insula demonstrated an increasing response to emotional faces with increasing age in the low PLEs group, and a decreasing response over time in the high PLEs group. The change in parahippocampal/amygdala and insula responses during the perception of emotional faces in adolescents with high PLEs between the ages of 14 and 19 suggests a potential ‘aberrant’ neurodevelopmental trajectory for critical limbic areas. Our findings emphasize the role of the frontal and limbic areas in the aetiology of psychotic symptoms, in subjects without the illness phenotype and the confounds introduced by antipsychotic medication.

## Introduction

*Psychotic-like experiences* (PLEs) describe transitory phenomena that, if they persist, can lead to clinically relevant symptoms with functional impairment^1^. A *developmental model of psychosis* describes transitory symptoms, such as PLEs and attenuated psychotic symptoms (psychosis proneness) becoming abnormally resilient (persistence) and subsequently clinically relevant (symptoms of clinical psychosis and impairment). This model is in line with the contemporary view that psychotic symptoms are not “all-or-nothing” pathological phenomena but rather fall within a spectrum ranging from normal, transient PLEs to pervasive psychotic symptoms, conceptualised as the *continuum model of psychosis*^2^. This view is supported by the high prevalence rates of sub-clinical delusional or hallucinatory experiences in the general population (10% and 30%), which is substantially higher than the prevalence rate of psychotic disorders^3^. In support of this model, structural brain abnormalities evident in adults with psychosis are also observed in adolescents with psychotic symptoms and free from the confound of medication effects^4^.

*Late adolescence* is a critical neurodevelopmental period, flagged by ongoing changes in brain structure and neural circuitry. These changes can be principal for the development of psychosis, as supported by the observation that the typical age of its onset occurs between late adolescence and early adulthood. The development of clinical psychosis can be a lengthy process, and individuals at increased risk, often referred to as the *At-Risk-Mental-State* (ARMS), can be characterised by using appropriate tools (i.e. the Comprehensive Assessment of the At-Risk Mental State, CAARMS, a structured questionnaire administered by clinicians, aiming to detect a variety of attenuated psychotic symptoms). Though conversion rates widely vary, approximately 20–35% of individuals aged 12–35 years who meet clinical criteria for a psychosis risk syndrome convert to clinical psychosis within two years^5^. During adolescence, particular epidemiological factors confer an increased risk for the future development of clinical psychosis; as shown by a single and non-replicated longitudinal study, the presence of various degrees of psychotic symptoms at age 11 years and cannabis use by the age of 15 years increases the likelihood of experiencing symptoms of schizophrenia in adulthood^6^.

PLEs offer a useful, non-clinical phenotype to study the spectrum of psychotic presentations^7^ with the advantages of a lack of exposure to the effects of both the illness and antipsychotic medication, and the possibility to study larger, non-clinical populations. Consequently, there is scope for the early detection and characterisation of PLEs in adolescence; the Community Assessment of Psychic Experiences Questionnaire (CAPE) can be the tool of choice, as the most widely used community assessment of PLEs^8^.

Patients with schizophrenia have long been recognised as having *deficits in emotional processing*, manifested as dysfunction in the domains of emotional expression, emotional experience (including hedonic responses) and emotional recognition (the ability to accurately identify and interpret emotions from other sources, including facial expressions)^9^; this has been attributed to aberrant information processing within a broader neural circuit, including the prefrontal cortices and the amygdala. A meta-analysis of neuroimaging studies of face processing comparing schizophrenia patients with healthy controls, showed patients demonstrating statistically significant under-recruitment of amygdalae and related regions including the parahippocampal cortex in response to aversive emotional material^10^; reduced activation was also evident in the right ventrolateral prefrontal cortex (VLPFC) and the right fusiform face area (FFA)^11^. Aberrant activation of the amygdalae and related regions including the parahippocampal cortex during processing of emotional stimuli was reflected in an inability to appropriately utilise and contextualise social cues. This deficit would naturally lead to the development of inappropriate suspicion, persecutory beliefs, with further impairment of interpersonal functioning^12^. Indeed, the insular cortices, caudate^13^ and the right parahippocampal gyrus^14^ demonstrate attenuated activation during facial emotion processing in patients with schizophrenia.

As PLEs offer a unique opportunity to study of psychosis unobstructed by the confounders of antipsychotic medication, and there is an observed association between schizophrenia and emotion processing, a reasonable step to take is to investigate a similar link between PLEs and emotion processing, employing a relevant neuroimaging task. There are only a few studies using neuroimaging during emotion processing tasks to investigate the neural basis of PLEs. A study of undergraduate students showed high CAPE scorers manifested greater activation in a number of prefrontal regions during reappraisal (reinterpretation of negative pictures); while the amygdala response to negative stimuli was decreased through reappraisal in the low scorers; functional connectivity analysis revealed lower prefrontal-amygdala coupling in high PLEs subjects^15^. Most recently, a functional Magnetic Resonance Imaging (fMRI) study employing an adolescent sample of subjects with PLEs (n = 27) at age 14 demonstrated *increased* hippocampus/amygdala/middle temporal gyrus and cerebellar activation during processing of neutral faces relative to subjects with low degree or absence of PLEs^16^.

The aim of the study was to examine whether PLEs in adolescence are associated with altered activation of frontal and limbic areas of a brain network, which have shown perturbed activation during facial emotion processing in subjects with psychosis. Three hypotheses were evaluated: the presence of elevated PLEs in late adolescence (age 19) will be associated with decreased activation of the amygdala/parahippocampal cortex, and associated network including prefrontal, insular and caudate regions during a facial emotion task^15,16^; the activation within these areas will differentially vary between high and low PLEs groups over the time between early (age 14) and late (age 19) adolescence; and the presence of increased PLEs in late adolescence will be associated with deficits in behavioural performance during risky and affective decision-making tasks^17^.

## Methods

### Participants and settings

As discussed previously (see acknowledgments), neuroimaging and clinical data of healthy adolescents were obtained from the IMAGEN database, https://imagen-europe.com/ accessed in August 2018. The IMAGEN study received ethical approval by the ethics research committees of the academic centres at which the study was conducted (London, Nottingham, United Kingdom and Dublin, Ireland; Paris, France and Berlin, Hamburg, Mannheim and Dresden, Germany). Ethical approval was obtained from the local ethics committee at each study site (**London**: Institute of Psychiatry; **Nottingham**: University of Nottingham; **Dublin**: Trinity College Dublin; **Paris**: National Institute of Health and medical Research (INSERM); **Berlin**: Charité University Berlin; **Hamburg**: University Medical Center Hamburg-Eppendorf; **Mannheim**: Central Institute of Mental Health Mannheim; **Dresden**: Technical University Dresden (TUD)).

All research methods were carried out in accordance with local guidelines and regulations. All adult participants provided written informed consent; minors provided oral informed consent and written informed consent was obtained by their parents or legal guardians. For further information please see our guidelines using the following link: www.nature.com/srep/policies/index.html#experimental-subjects.

The present study was conducted from January 1, 2016 to January 1, 2017, from anonymised data. Our study involved no information, either data or images, that could lead to the identification of participants. No such information is presented in the present publication. Access to IMAGEN database for the conduction of the present study did not require a separate informed consent; a waiver was applied owing to fully anonymised data. We used data collected at age 14 and 19. A total of 1,434 adolescents were initially selected based on quality controls and completeness of their behavioural and neuroimaging datasets. Two subgroups were assessed at ages 14 and 19 years. Those who scored at either high or low PLEs (based on the upper and lower deciles) on the CAPE-42 items instrument (see below) at age 19 were included in the analysis. The epidemiological features of our sample are described in Table 1, the exclusion criteria are listed in the supplement. Our sample included healthy individuals and the presence of major medical, neurological, developmental or psychiatric conditions, as well as pregnancy complications, was exclusionary; similarly, participants who developed a major psychiatric disorder during the study, were excluded from further follow-up.

**Table 1:** Participant Characteristics and CAPE-42 Score Stratification.

	CAPE TOTAL HIGH 10% (n = 149)			CAPE TOTAL LOW 10% (n = 149)		
	Mean	SE	SD	Mean	SE	SD
Gender (Male %)	33.60%			56.40%		
Handedness (R %)	85.90%			82.60%		
Age BL (y)	14.47	0.03	0.39	14.43	0.03	0.38
Age FU (y)	19.02	0.06	0.76	18.98	0.06	0.74
Wisc Verbal Score (BL)	111.15	1.32	15.66	106.72	1.27	15.24
Wisc Performance Score (BL)	108.49	1.33	15.79	105.2	1.20	14.42
Adrs Total Score (FU)	15.89	0.24	2.96	19.7	0.06	0.72
Audit Total Score (FU)	7.5	0.44	5.34	5.26	0.32	3.87
Dast Cannabis Total Score (FU)	1.59	0.21	2.52	0.54	0.08	1.02
Cape Total Score (FU)	111.64	1.74	21.26	9.54	0.39	4.75
Cape Positive Symptoms Score (FU)	33.09	1.27	15.48	3.23	0.24	2.98
Cape Bizarre Delusions Score (FU)	13.17	0.97	11.82	0.37	0.09	1.09
Cape Social Delusions Score (FU)	19.91	0.54	6.57	2.87	0.21	2.61
Cape Negative Symptoms Score (FU)	46.37	0.93	11.37	2.22	0.21	2.61
Cape Depressive Symptoms Score (FU)	32.18	0.61	7.49	4.09	0.21	2.54

WISC: Wechsler Intelligence Batter for Children^38^; the average score is 100; higher scores indicate higher than average intelligence and lower scores indicate lower than average intelligence.

ADRS: Adolescent Depression Rating Scale^39^; scores range from 0 to 60; higher scores indicate higher levels of adolescent depression.

AUDIT: Alcohol Use Disorders Identification Test^40^; scores range from 0 to 40; higher scores indicate greater levels of alcohol abuse.

DAST: Drug Abuse Screening Test^41^; scores range from 0 to 10; higher scores indicate greater levels of cannabis abuse.

CAPE: Community Assessment of Psychic Experiences Questionnaire^7^; scores range from 0 to 294; higher scores indicated elevated presence of attenuated psychotic symptoms.

SD: Standard Deviation; SE: Standard Error.

### Measures

The CAPE-42 questionnaire^7^ was used as a measure of PLEs in our adolescent population at age 19 years. Based on the PDI-21(21-items Peters *et al*. Delusional Inventory)^18^ and PDI-40 (40-items Peters *et al*. Delusional Inventory)^19^, the CAPE-42 is a self-administered tool, including 42 items that are grouped in three dimensions: positive, negative, and depressive. Each item is scored for frequency and severity in a scale from 0 to 7; total scores range from 0 to 294. Higher scores indicate higher burden of symptoms found in the psychosis prodrome.

The Cambridge Neuropsychological Test Automated Battery (CANTAB) includes highly sensitive, precise and objective measures of cognitive function^20^. Our study focused on the Affective Go-NoGo Task (AGN), providing an assessment of the information processing biases for positive and negative stimuli; this task was chosen as a proxy for ‘hot’ cognition (cognitive functions mostly influenced by the individual’s emotional state), as related to the inhibitory/affective function of frontal and limbic areas of the brain. Participants completed the AGN at both age 14 and 19.

In the Face Task (FT) volunteers are asked to passively watch short black and white video clips presenting faces with neutral and angry expressions as well as control non-biological motion stimuli (concentric circles)^21^. In our study, we focused on the contrasts angry faces vs control stimuli, angry faces vs neutral faces and angry and neutral faces vs control stimuli, as previously described in the IMAGEN literature^22,23^. Additional literature identified common variance in response to ambiguous facial expression^23^. Participants completed the FT at both age 14 and 19.

Details of the CAPE-42, CANTAB AGN and FT are described in the supplement.

### Stratification of the sample

We defined two subgroups with high or low PLEs, based on total CAPE-42 scores at age 19 years. Because the CAPE-42 total scores did not follow a normal distribution (eFig. 1), we selected participants with high and low scores using the upper and lower deciles. This distinction resulted in 149 adolescents in the high PLEs group and 149 in the low PLEs group. The high group CAPE-42 total score ranged from 91 to 182, corresponding to a standardised (per item) score range of 2·17 to 4·33. Cut-off levels in the region of 2.0 per CAPE-42 item (transposed to match our rating conventions) provide adequate positive predictive value for transition to psychosis^24^; based on our stratification, this level formed the lower bound of the high PLEs group. The two groups were equivalent for handedness, age and IQ.

### fMRI acquisition and analysis

The standardised scanning parameters were selected to be compatible and implementable at all sites and scanners. A full description of the scanning protocols, cross-site standardization and quality checks, and pre-processing of resulting data are provided elsewhere^25^ and also listed in the supplement.

### First-level analysis

Three within-subject contrasts reflecting core face emotional processing, was selected for investigation:

[Angry Faces] − [Control Stimuli];

[Angry Faces] − [Neutral Faces];

[Angry + Neutral Faces] − [Control Stimuli].

Control stimuli were non-facial motion stimuli. The contrasts were designed to separate emotional salient facial stimuli from neutral stimuli, and facial from non-facial stimuli, providing maximum differentiation, and were previously used in the IMAGEN population^22^. The fMRI scans were conducted at ages 14 and 19 years and were analysed using SPM12 (Statistical Parametric Mapping; http://www.fil.ion.ucl.ac.uk/spm Wellcome Trust Centre for Neuroimaging).

### Second-level analysis

Preliminary whole-brain analysis revealed no statistically significant correlations between brain activation levels and CAPE-42 total scores in the overall sample of 1,434 adolescents; as a consequence, further analysis focused only on high and low CAPE-42 scorers and task-related regions of interests for the chosen contrast were collapsed across the high and low groups. This approach provided an unbiased estimate of the activation, as the group average positive/negative mask is orthogonal to high > low or low > high masks and is also supported by the literature on functional localizers^26^. As multiple ROIs resulted in this step, we performed a retrospective Bonferoni correction of the statistical significance threshold, at a contrast level.

We have extracted mean brain activation contrasts (parameter estimates) for all the identified ROIs, at the ages of 14 and 19. In a factorial analysis (Mixed Model 2-way ANOVA), the main effects of *time*, *group* and the interaction of *time x group* on brain activation levels were examined by employing a 2-way analysis of variance, with group as fixed and subject as random effects. Any main effects or interactions were further examined by post hoc paired and unpaired, 2-tailed *t* tests, with *P* < 0·05 as the statistically significant threshold. Additional exploratory analyses are also reported.

## Results

Two groups of adolescents (high PLEs, n = 149, 50 [33·6%] male; low PLEs, n = 149, 84 [56·4%] male) were compared at ages 14 and 19 years. The two groups were equivalent for handedness, age and IQ.

### Results of fMRI analysis

**Figure 1:**
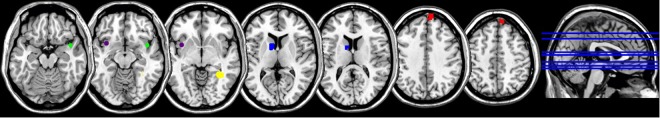
ROIs showing differences in brain activation between low and high PLEs groups. Coronal View; Horizontal Axis: Left = Left, Right = Right; Vertical Axis: Up=Superior, Down=Inferior. Colour Coding. Age 14-[42 8 −14]: Right Insular Cortex (H > L) [shown in green]. [−36 11 −5]: Left Insular Cortex (L > H) [shown in purple]. Age 19-[−12 5 10]: Left Caudate Body (L > H) [shown in blue]. [6 65 31]: Right Superior Frontal Gyrus (L > H) [shown in red].[33 −46 −5]: Right Parahippocampal Gyrus/Amygdala (L > H) [shown in yellow].

Five regions of interest (ROIs) based on previous studies of facial emotion processing in psychosis and UHR for psychosis^15,16^ were used; the prefrontal cortices, the temporal cortices, the limbic brain and the striatum were included for factorial analysis. The ROIs Montreal Neurological Institute (MNI) space coordinates were: left insular cortex (−36 11 −5), right insular cortex (42, 8, −14), left caudate body (−12, 5, 10), right superior frontal gyrus (6, 65, 31) and right parahippocampal gyrus/including the amygdala (33, −46, −5). One ROI (−36 11 −5) arose from the *angry vs neutral faces* contrast, while all remaining ROIs activated in the *angry faces vs control stimuli* contrast; no relevant activations were noted in the *angry plus neutral faces vs control stimuli* contrast. Only the right insular cortex ROI (42, 8, −14) did not survive a retrospective Bonferoni correction for multiple testing (p = 0.016 given a revised threshold at 0.01). Figure 1 provides a visual representation of the ROIs; eTable 1 in the Supplement reports the results of the functional ROI brain analysis.

### Left insular cortex (−36 11 −5)

**Figure 2:**
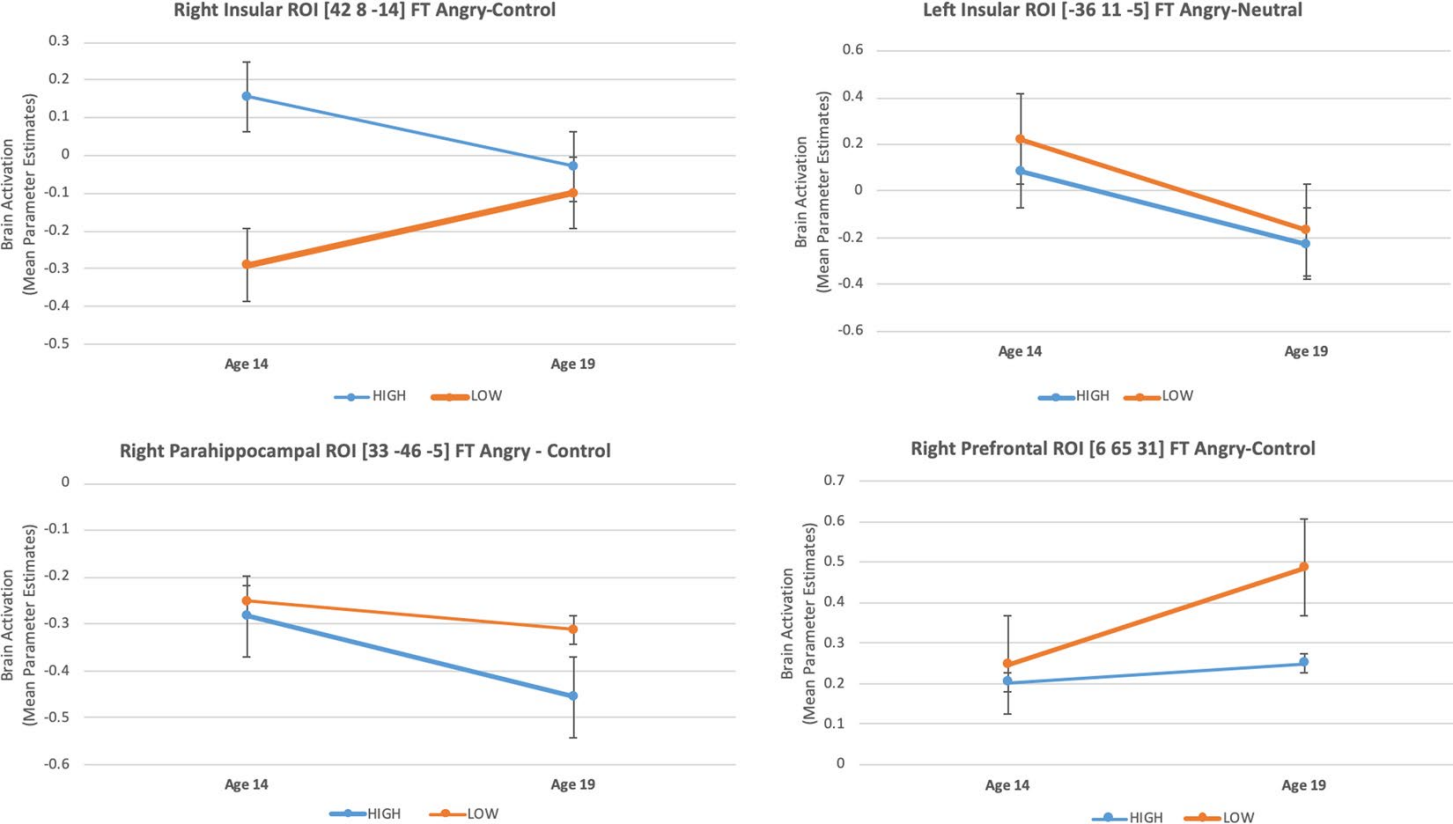
Mean Brain Activation (Parameter Estimates) at age 14 and 19 for Right Prefrontal ROI [6 65 31], Right and Left Insular ROIs [42 8 −14] & [−36 11 −5] and Right Parahippocampal ROI [33 −46 −5]; statistically significant changes at p = 0.05 level: for the low PLEs group only in [6 65 31] and [42 8 −14]; for the high PLEs group only in [33 −46 −5]; for both the high and low PLEs groups in [−36 11 −5]; SE bars are displayed.

There was a significant main effect of *group* (*F*_1,227_ = 4·5, *P* = 0·03), which was driven by decreased activation in the high PLEs group compared to the low PLEs group at *age 14* (*t* = −2·1, *P* = 0·04). There was also a significant main effect of *time* (*F*_1,227_ = 64·7, *P* < 0·0001) which was driven by a *decrease* in activation from *age 14 to age 19 years*, (high group *t* = 4·9 *P* < 0·0001; low group *t* = 6·4, *P* < 0·0001). Figure 2, Table 2, eTable 2, eTable 3.

**Table 2:** Mean Brain Activation Contrast Parameter Estimates Factorial Analysis.

	Type III Sum of Squares	df	Mean Square	F	Sig.	r
**Right Insular ROI [42 8 −14] Brain Activation**						
TIME	0.002	1	0.002	0.002	0.97*	0.003
GROUP	3.92	1	3.92	**19.52**	**<0.0001**	0.28
TIME * GROUP	8.23	1	8.23	**7.46**	**0.01**	0.18
Error (TIME)	253.77	230	1.10			
Error (GROUP)	46.2	230	0.20			
**Left Insular ROI [−36 11 −5] Brain Activation**						
TIME	13.99	1	13.99	**64.75**	**<0.0001**	0.47
GROUP	1.11	1	1.11	**4.56**	**0.034**	0.14
TIME * GROUP	0.21	1	0.21	0.96	0.33*	0.06
Error (TIME)	49.05	227	0.22			
Error (GROUP)	55.46	227	0.24			
**Left Caudate ROI [−12 5 10] Brain Activation**						
TIME	0.39	1	0.33	1.71	0.19*	0.09
GROUP	0.18	1	0.18	3.04	0.08*	0.14
TIME * GROUP	0.73	1	0.73	3.20	0.07*	0.12
Error (TIME)	52.78	230	0.23			
Error (GROUP)	13.99	230	0.06			
**Right Prefrontal ROI [6 65 31] Brain Activation**						
TIME	4.83	1	4.83	**6.98**	**0.01**	0.17
GROUP	1.15	1	1.15	**4.21**	**0.04**	0.13
TIME * GROUP	2.19	1	2.15	3.17	0.08*	0.12
Error (TIME)	159.10	230	0.69			
Error (GROUP)	62.56	230	0.27			
**Right Parahippocampal ROI [33 −46 −5] Brain Activation**						
TIME	3.17	1	3.17	**12.11**	**0.001**	0.22
GROUP	0.45	1	0.45	**4.90**	**0.03**	0.14
TIME * GROUP	0.72	1	0.72	2.761	0.1*	0.11
Error (TIME)	60.25	230	0.26			
Error (GROUP)	21.36	230	0.09			

Abbreviations df: degrees of Freedom; F: F-ratio; (*): not statistically significant at a p = 0.05 level; r: Pearson’s correlation coefficient.

### Right insular cortex (42, 8, −14)

There was a significant interaction effect of group*time (*F*_1, 230_ = 7·4, *P* = 0·01) driven by a differential change in the groups’ brain activation from *age 14 to age 19*, showing a relative (non-significant) decrease in the high PLEs group, and an increase in the low PLEs group (*t* = −2·0, *P* = 0·04). Additional exploratory post hoc analysis exhibited increased activation of the high PLEs group compared to the low PLEs group at *age 14* (*t* = 5·2, *P* < 0·0001). Figure 2, Table 2, eTable 2, eTable 3.

### Left caudate body (−12, 5, 10)

There were no significant effects of group*time, nor any main effects of *group* or *time*. Additional exploratory analysis exhibited decreased activation in the high PLEs group compared to the low PLEs group *at age 19* (*t* = −2·588, *P* = 0·01). Figure 2, Table 2, eTable 2, eTable 3.

### Right superior frontal gyrus (6, 65, 31)

There was a main effect of *group* (*F*_1, 230_ = 4·2, *P* = 0·04), which was driven by decreased activation in the high PLEs group compared to the low PLEs group at *age 19* (*t* = −2·3, *P* = 0·02). There was also a main effect of time (*F*_1, 230_ = 6·9, *P* = 0·01), which was driven by an *increase* in activation from *age 14 to age 19 years*, post hoc analyses showed this was significant only for the low PLEs group (*t* = −3·3, *P* = 0·001). Figure 2, Table 2, eTable 2, eTable 3.

### Right Parahippocampal Gyrus/Amygdala (33, −46, −5)

There was a main effect of group (*F*_1, 230_ = 4·9, *P* = 0·03), which was driven by increased activation in the low PLEs group compared to the high PLEs group at *age 19* (*t* = −2·8, p = 0·005). There was a main effect of time (*F*_1, 230_ = 12·1, *P* = 0·001), which was driven by a *decrease* in activation from *age 14 to age 19 years*, post hoc analyses showed this was significant only the high PLEs group (*t* = 4·3, *P* < 0·0001). Figure 2, Table 2, eTable 2, eTable 3.

### Results CANTAB measures analysis

There was a main effect of time on AGN Total Omissions for both positive (*F*_1, 159_ = 58·8, *P* < 0·0001] and negative stimuli (*F*_1, 159_ = 50·2, *P* < 0·0001]; this was driven by a decrease, signifying improved performance, from *age 14 to 19*, across both the high and low PLE groups (high - AGN positive: *t* = 4·5, *P* < 0·0001; AGN negative: *t* = 3·7, *P* < 0·001 and low - AGN positive: *t* = 6·3, *P* < 0·0001; AGN negative: *t* = 6·4, *P* < 0·0001). Additional exploratory analysis revealed that the high PLEs group scored lower than the low PLEs group on AGN Total Omissions for both positive and negative stimuli at age *14 years*, which indicates a better performance at inhibitory processing in the high versus the low PLEs group. Table 3, eTable 4, eTable 5.

**Table 3:** CANTAB Measures Factorial Analysis.

Factorial Analysis, Mixed Model 2-way ANOVA							
CANTAB Variable	Factors	Type III Sum of Squares	df, dfR	Mean Square	*F* value	***P*** **value**	***r*** **value**
AGN Total Omissions Negative	GROUP	73.82	1, 16	73.82	2.5	0.12*	0.12
	TIME	3006.21	1, 16	3006.21	**50.24**	**<0.0001**	0.49
	GROUP * TIME	144.6	1, 16	144.6	2.42	0.12*	0.12
AGN Total Omissions Positive	GROUP	77.1	1, 16	77.1	2.92	0.1*	0.13
	TIME	3423.72	1, 16	3423.72	**58.78**	**<0.0001**	0.52
	GROUP * TIME	77.33	1, 16	77.33	1.33	0.25*	0.09

Abbreviations,

AGN Total Omissions Negative/Positive: Affective Go-NoGo Task, total number of missed responses to targets in the blocks specified by the value of target type (negative, positive); CANTAB: Cambridge Neuropsychological Test Automated Battery;

df: degrees of Freedom; dfR: df(Error) F: F-ratio; r: Pearson’s correlation coefficient: (*): not statistically significant at a *P *= 0.05 level.

### Correlations between CANTAB measures and brain activation levels

At age 14, brain activation levels at the right insular region (42, 8, −14) showed negative correlation with AGN Total Omissions for both positive and negative stimuli (AGN positive: *r* = −0·181, *P* = 0·01; AGN negative: *r* = −0·219, *P* = 0·002). At age 19, brain activation levels at the same region showed positive correlation with AGN Total Omissions for negative stimuli (*r* = 0·163, *P* = 0·03).

## Discussion

The main findings of our analysis are summarised in Table 4. Overall, the group with lower CAPE scores showed increased activation, compared to the group with higher CAPE scores in the left insula (age 14), the left caudate, the right prefrontal and the right parahippocampal areas (age 19); the high CAPE score group exceeded the low CAPE score group in activation only in the right insula (age 14).

**Table 4:** Overall Results of Mean Brain Activation and CANTAB Measures Factorial and Exploratory Analysis.

Brain ROI/CANTAB measure		Exploratory Analysis (significant effects)		Factorial Analysis		
				Age 14	Change between 14 and 19	Age 19
Brain ROIs	Left Insular Cortex **[−36 11 −5]**	**Group, Time**	**L** > **H**	**H ↓**	**L ↓**	
	Right Insular Cortex **[42 8 −14]**	Group, Group*Time	**H** > **L**	**H** ↓ NS	**L ↑**	
	Left Caudate Body **[−12 5 10]**					**L** > **H**
	Right Superior Frontal Gyrus **[6 65 31]**	**Group, Time**		**H** ↑ NS	**L ↑**	**L** > **H**
	Right Limbic Cortex **[33 −46 −5]**	**Group, Time**		**H ↓**	**L** ↓ NS	**L** > **H**
CANTAB measure	AGN Total Omissions, Positive Stimuli	**Time**	**L** > **H**	**H ↓**	**L ↓**	
	AGN Total Omissions, Negative Stimuli	**Time**	**L** > **H**	**H ↓**	**L ↓**	

**H**: High PLEs Group; **L**: Low PLEs Group;

**↑**: Increase in brain activation or CANTAB score (statistically significant);

↑ NS: Increase in brain activation or CANTAB score (statistically non-significant);

**↓**: Decrease in brain activation or CANTAB score (statistically significant);

↓ NS: Decrease in brain activation or CANTAB score (statistically non-significant).

19-year-old subjects with increased CAPE scores, indexing higher PLEs (measured only at age 19), demonstrated attenuated activation of the right superior frontal gyrus, the right parahippocampal gyrus/amygdala and the left caudate, compared to their peers with lower CAPE scores, indexing lower PLEs – when examined using fMRI during a facial emotion perception test. These regions form components of a broader neural circuit responsible for social cognition and behaviour, encompassing the amygdalae, the VMPFC, the cingulate cortex, somatosensory cortices, the fusiform gyrus and the superior temporal sulcus^27^. Misidentification of facial expressions represent a crucial deficit in this process, and as observed extensively in the schizophrenia literature, can give rise to impaired social interactions and the subsequent elaboration of paranoid and persecutory beliefs. The right parahippocampal gyrus, was also observed to show increased activation in controls, compared to schizophrenia patients, during the perception of fearful faces^14^. In our study, we noticed increased activation in the right parahippocampal gyrus including foci within the amygdala in the low PLEs group, at the age of 19, during perception of angry faces. These observations provide neuroimaging evidence supporting the *extended psychosis phenotype* encompassing preclinical and clinical presentations.

While the low PLEs group showed a regular trajectory of increasing activation over time in the right prefrontal and right insular cortices with brain maturation, the high PLEs group demonstrated decreased activation of the right parahippocampal/amygdala and left insular activation over the same time. This normal increase in frontal activation from age 14 to 19 years is thought to reflect a consequence of the increasing prefrontal cortical control evidenced by the routine improvement in behavioural impulsivity measures to affective stimuli assessed by the Affective Go-NoGo task. Besides, lack of change (or more precisely, a non-significant decrease) in the right insular activation for the high PLEs group between the ages of 14 and 19 results in a *‘normalisation’* of an overactivation at age 14, as the low PLEs group demonstrates a significant increase in right insular activation between the two timepoints. Our longitudinal analysis confirmed that in the high PLEs group, brain activation of a parahippocampal area including foci within the amygdala showed an attenuation between the ages of 14 and 19; the same area also demonstrated significantly decreased activation in the low PLEs group, at the age of 19. This variation could potentially underpin an *‘aberrant’ developmental process*, leading to under-recruitment of critical limbic areas during perception of angry faces, introducing a risk for the future emergence of psychosis. Between the ages of 14 and 19, the high PLEs group compared to the low PLEs group showed no significant change in activation of the prefrontal cortex [6 65 31]; the same area remained under-active at the age of 19 in the high PLEs group, which could represent a *residual deficit* in frontal activation.

Hypoactivation in *frontal areas* might represent a *trait* (pattern of brain activation which persist along various timepoints) in the development of psychosis, as it appears in all phases of the continuum, from *preclinical* to *schizophrenia*. *Frontal hypoactivation* or *‘hypo-frontality’* has been extensively researched in schizophrenia, in relation to a variety of behaviours, including motivation, executive functions and psychotic symptomatology^28^. Another position is that during working memory tasks, patients with schizophrenia can either recruit more extended prefrontal resources, or fail to sustain recruitment of adequate prefrontal areas, compared to controls, which eventually results in poorer behavioural outcomes^29^. Functional dysconnectivity of fronto-striatal circuitry may represent a risk phenotype for psychosis. First-episode psychosis is associated with pronounced dysregulation of cortico-striatal systems, characterized most prominently by hypo-connectivity of dorsal and hyper-connectivity of ventral fronto-striatal circuits^30^. A meta-analysis of neurofunctional correlates of vulnerability to psychosis revealed hypoactivation of DLPFC and VLPFC as the most common finding in Ultra-High Risk (UHR) and First-Episode Psychosis (FEP) populations^31^. Our cross-sectional results indeed revealed hypoactivation of prefrontal [6 65 31] and striatal [−12 5 10] regions in the high PLEs group at age 19, a finding consistent with perturbed fronto-striatal connectivity.

Aberrant activation of the *amygdala* during a face recognition is probably one of neuroimaging hallmarks of psychosis. In our study, we identified a large cluster centred in the right parahippocampal gyrus, including the amygdalae, hippocampal, and parahippocampal areas, which demonstrated a peak activation at a right parahippocampal region, [33–46], which demonstrated significantly *lower* activation for the high PLEs group at the age of 19. A study employing a similar IMAGEN sample as ours revealed that subjects with PLEs demonstrated, among other findings, *increased* hippocampus/amygdala activation during processing of neutral faces, compared to controls^16^. It is of note however that the authors of this study employed a smaller sample (n = 27), assessed PLEs at age 14 only and used a less extended questionnaire focusing on perceptual abnormalities and delusional thoughts. It’s relevant that we are assessing a non-clinical group of subjects; thus, our findings are likely to correspond to preliminary brain alterations in a trajectory toward psychosis and predate changes that are later seen more consistently across the psychosis continuum.

Our finding of lower left caudate activation at age 19 in the high PLEs group, is of interest. A similar finding in this region has been reported by another study, showing that UHR individuals had decreased left caudate activation during processing of prosodic voice with negative emotional valence, compared to increases activation in controls^32^.

The *neuropsychological assessment* showed a decrease in AGN scores from age 14 to age 19 in both the high and low PLEs groups, which indicates an improvement in affective/inhibitory control. This change corresponds to findings of decreased limbic activation [33, −46, −5] from age 14 to age 19 in the high PLEs group, and increased frontal activation [6, 31, 65] from age 14 to age 19 in the low PLEs group. Knowing that processing of emotional material is largely mediated by limbic areas of the brain, while the frontal lobes can exert a top-down inhibitory control in subcortical areas, here we detect a dual pattern of achieving improved emotional processing, by either increasing ‘higher’ cortical control (high PLEs group) or decreasing ‘lower’ limbic activation (low PLEs group). Hyperactivation in the limbic system can account for an inability to regulate emotions, and as discussed earlier, emotion regulation difficulties can lie at the core of vulnerability to psychosis^15^.

Interestingly, the high PLEs group exhibited a lower rate of omissions in the AGN task, indexing increased affective/inhibitory control, compared to the low PLEs group at age 14. This observation cannot be accounted by the limited corresponding findings of increased right insular activation [42 8 −14] and decreased left insular activation [−36 11 −5] in the same group at the same age; neither can be interpreted within the boundaries of the continuum model of psychosis. However, similar inconsistencies between neuroimaging findings and cognitive-behavioural measures point towards the differences in sensitivity of the two approaches to detect an underlying brain process^33^.

### Limitations

There is a number of *limitations* in our study. Firstly, we selected CAPE upper and lower deciles to stratify our sample, as these correspond to cut-off levels roughly equivalent to those associated with psychosis proneness in literature (detailed in the section describing the stratification of our sample). The decision to dichotomize our population was mainly driven by the need to identify the most/least prone to psychosis individuals in a naturalistic sample, given that CAPE-42 scores were still moderate in it. Despite the limitations introduced by the use of dichotomization, this was deemed as the most appropriate method to explore the extremes of the psychosis continuum in our naturalistic sample. Nevertheless, we eventually ended up with a high PLEs group having a still low average total score of 112, considering the full potential range of scoring (0–294). In addition, as we stratified our sample retrospectively, based on CAPE-42 scores at age 19 year, and in light of the absence of available scores at age 14 years or intermediate ages, it was impossible to observe the evolution of PLEs between the two timepoints. We have rather arbitrarily viewed the high-PLEs phenotype as a representative for the prodrome for psychosis; however, PLEs can have multiple clinical outcomes, thus leading to a variety of psychopathologies, other than florid psychosis *or* can also be associated with non-clinical phenotypes as manifested by a mean lifetime prevalence of>5% of psychotic experiences in the general population^34^. Obviously, the lack of transition to psychosis data restricts the use of our high PLEs group as a measure of the UHR population. There was a greater representation of males in our low vs high PLEs groups; despite male predominance being a common epidemiological finding in this clinical field^35^, this may impede the generalisability of our results. Moreover, we did not control for gray matter volumetric changes.

In our study we focused on contrasts involving angry faces aiming at emotional aspects of faces’ processing. It would be of interest for future studies to analyse contrasts allowing the detection differences in baseline activation (i.e. neutral faces vs control stimuli) which can be suggestive of an aberrant salience processing^36^.

## Conclusions

In a previous study from the same IMAGEN sample, we provided evidence for a consistent increase in prefrontal activation during reward feedback, in the high PLEs group, between the ages of 14 and 19, which was attributed to a compensatory cognitive control mechanism^37^. The current study revealed functional alterations in parahippocampal/amygdala and insula responses during the perception of emotional faces in adolescents with high PLEs between the ages of 14 and 19 suggests a potential ‘aberrant’ neurodevelopmental trajectory for critical limbic areas. The two studies complement each other, while employing two different cognitive paradigms to examine the neural basis of PLEs in adolescence. Our findings emphasize the role of the frontal and limbic areas in the aetiology of psychotic symptoms in line with a continuum model, in a sample of subjects without the illness phenotype and the confounds introduced by antipsychotic medication.

## Supplementary information

Supplementary Material.
