# Supplementary material for: Examination of the neural basis of psychotic-like experiences in adolescence during processing of emotional faces
Source: Sci Rep. 2020 Mar 20;10:5164. doi: 10.1038/s41598-020-62026-7 (PMC7083946; doi:10.1038/s41598-020-62026-7)
Supplement: Supplementary file 1 — Supplementary Material. [file 41598_2020_62026_MOESM1_ESM.docx]

# Supplementary Material

**Examination of the neural basis of psychotic-like experiences in adolescence during processing of emotional faces.**

Dr Evangelos Papanastasiou, MD, MSc, PhD; Dr Elias Mouchlianitis, MSc, PhD; Dr Dan W. Joyce, BSc, PhD, MBBS, MRCPsych; Prof Philip McGuire, MB ChB, MD, PhD, FRCPsych, FMedSci; Ms Celia Boussebaa, BSc Candid. ; Prof Tobias Banaschewski, PhD; Dr Arun L.W. Bokde, PhD; Prof Christian Büchel, MD; Dr Erin Quinlan, PhD; Dr Sylvane Desrivières, PhD; Prof Herta Flor, PhD; Dr Antoine Grigis, PhD; Dr Hugh Garavan, PhD; Mr Philip Spechler; Prof Penny Gowland, PhD; Prof Andreas Heinz, MD; Dr Bernd Ittermann, PhD; Dr Marie-Laure Paillère Martinot, MD, PhD; Dr Eric Artiges, MD, PhD; Dr Frauke Nees, PhD; Dr Dimitri Papadopoulos Orfanos, PhD; Prof Tomáš Paus, MD, PhD; Prof Luise Poustka, MD; Ms Sabina Millenet, Dipl.-Psych.; Ms Juliane H. Fröhner, MSc; Prof Michael N. Smolka, MD; Dr Henrik Walter, MD, PhD; Dr Robert Whelan, PhD; Prof Gunter Schumann, MD, PhD; Prof Sukhwinder Shergill, MBBS, SFHEA, PhD, FRCPsych.

## IMAGEN Exclusion Criteria

| Pregnancy and birth | 1. Use of alcohol by the mother during pregnancy (>210 ml alcohol/week) |
| --- | --- |
|  | 2. Diabetes of the mother during pregnancy (onset before pregnancy, treated by insulin) |
|  | 3. Premature birth (< 35 weeks) and/or detached placenta |
|  | 4. Hyperbilirubinemia requiring transfusion |
| Medical History | 1. Type 1 diabetes |
|  | 2. Systemic rheumatological disorders |
|  | 3. Malignant tumours requiring chemotherapy |
|  | 4. Congenital heart defects or heart surgery |
|  | 5. Aneurism |
| Neurological conditions | 1. Epilepsy |
|  | 2. Bacterial Infection of CNS |
|  | 3. Brain tumour |
|  | 4. Head trauma with loss of consciousness >30 minutes |
|  | 5. Muscular/myotonic dystrophy |
| Developmental conditions | 1. Nutritional and metabolic diseases |
|  | 2. Major neuro-developmental disorders |
|  | 3. Hearing deficit requiring hearing aid |
|  | 4. Vision problems (strabismus, visual deficit not correctible) |
| Mental Health | 1. Treatment for schizophrenia, bipolar disorder |
|  | 2. IQ < 70 |
| MRI Contraindications | 1. Metal implants |
|  | 2. Electronic implants |
|  | 3. Severe claustrophobia |

## IMAGEN fMRI Acquisition Parameters

| **Sequence Parameter** | **Value** |
| --- | --- |
| No. of Volumes | 191 (MID), 202 (FT) |
| TR (ms) | 2200 |
| TE (ms) | 30 |
| ETL | 32 |
| NSA | 1 |
| Excitation flip angle (degrees) | 75 |
| 2D/3D | 2D |
| Voxel Size (mm) | 3.4 x 3.4 x 2.4 |
| Matrix size | 64^2^ |
| No. of slices/DDAs | 40/3 |
| FOV frequency (mm) | 218 |
| FOV phase (%) | 100% |
| Slice thickness (mm) | 2.4 |
| Slice gap (mm) | 3.4 |
| Slice orientation | Oblique (ACPC) |
| In-plane phase encode direction | Anterior-Posterior |
| Slice acquisition order | Sequential |
| Slice acquisition direction | Superior-Inferior |
| **Abbreviations**  **TR:** Repetition Time; **TE:** Echo Time; **ETL:** Echo Train Length; **NSA:** Number of Signal Averages (average number for each phase encoding step); **DDA:** Discarded or Disabled Acquisitions, Dummy Cycles; **FOV:** Field of View; **ACPC:** Anterior Commissure-Posterior Commissure Plane | |

## IMAGEN fMRI standardisation

fMRI was performed on 3T scanners from a range of manufacturers (Siemens, Munich, Germany; Philips, Best, The Netherlands; General Electrics, Chalfont St Giles, UK; Bruker, Ettlingen, Germany). A key challenge for the ability to pool data acquired on MR scanners of different manufacturers related to their variation in availability and implementation of particular image-acquisition techniques. To address this problem, for each technique, a set of parameters compatible with all scanners, particularly those directly affecting image contrast or signal-to- noise, was devised and held constant across sites. Where manufacturer-specific choices had to be made (for example the design of head coil), the best manufacturer-specific option was used at all sites with the same scanner type. Two quality control procedures were regularly implemented at each site: (a) The American College of Radiology phantom was scanned to provide information about geometric distortions and signal uniformity related to hardware differences in radiofrequency coils and gradient systems, image contrast and temporal stability, and a custom phantom was scanned for diffusion related parameters. (b) Several healthy volunteers were regularly scanned at each site to assess factors that cannot be measured using phantoms alone and at multiple sites to determine inter-site variability in structural and functional measures (for example, tissue contrast in raw MRI signal, tissue relaxation properties).

### The FACE task (FT)

**Blocks of videos showing faces neutral and angry faces.** Faces that turn from neutral to angry or stay neutral, are interspersed with blocks of control stimuli.

**Instructions in English**

In the following scanning session, we will ask you to perform different tasks and games which are so far unfamiliar to you. Therefore, we would like to explain these tasks and games first outside the scanner using this standard computer. Additionally, you have the opportunity to practice some of the tasks. Let’s start with a few simple tasks which target basic functions of the brain.

In this task you will be presented with short video clips showing faces with neutral and angry expressions as well as moving circles. Please watch them carefully and remember to lie as still as possible during this task.

### CANTAB Affective Go-NoGo Task (AGN)

### **
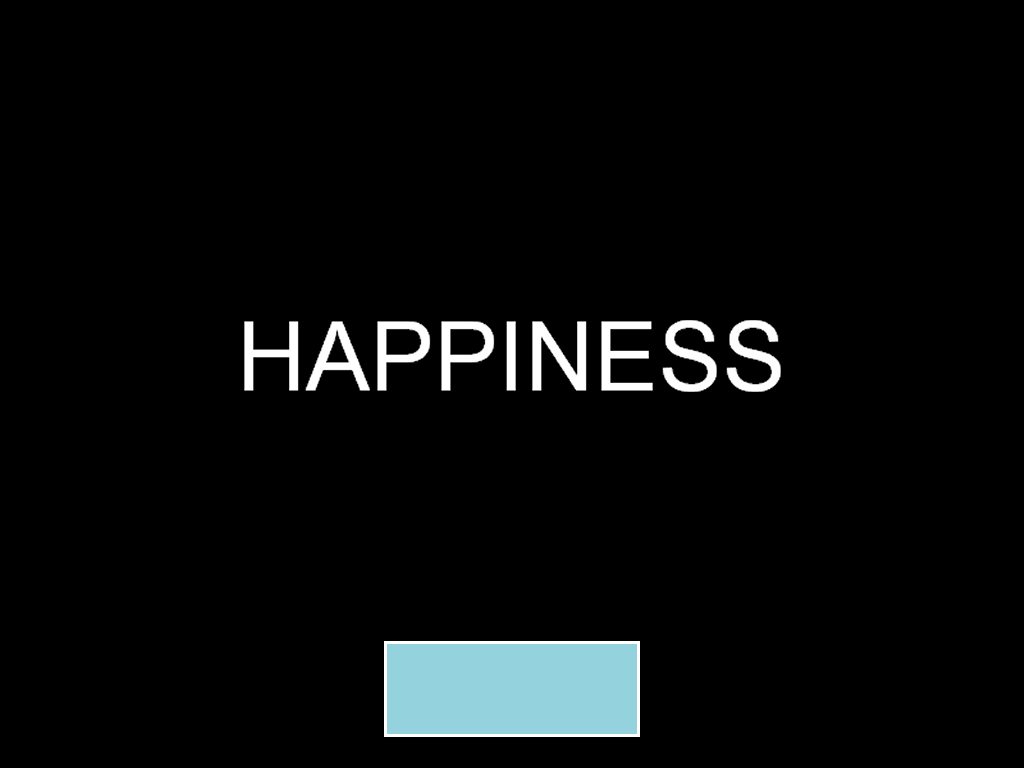
**

**Administration time:** Around 10 minutes, depending on level of impairment. The modified IMAGEN version lasts approximately 8 minutes.

**Task:** The test consists of several blocks, each of which presents a series of words from two of three different Affective categories: positive (e.g. joyful), anxiety-related (e.g. attack), depression-related (e.g. useless) and neutral (e.g. element). The subject is given a target category, as is asked to press the pad when they see a word matching this category.

**Test modes:** Six modes. Four using positive and negative stimuli only, two using positive, negative and neutral stimuli. The affective Go-NoGo task was supplemented by anxiety-related words and the total number of blocks were reduced to shorten the task. IMAGEN used this modified mode including positive, anxiety-related and depression-related and neutral stimuli.

**Outcome measures:** Several measures covering latency and errors of commission and omission.

**AGN Mean correct latency (positive):** Average reaction time for correctly identified positive stimuli.

**AGN Mean correct latency (negative):** Average reaction time for correctly identified negative stimuli.

**AGN Mean correct latency (neutral):** Average reaction time for correctly identified neutral stimuli.

**AGN Total commissions (positive):** Number of responses to distractors for positive stimuli.

**AGN Total commissions (negative):** Number of responses to distractors for negative stimuli.

**AGN Total commissions (neutral):** Number of responses to distractors for neutral stimuli.

**AGN Total omissions (positive):** Number of missed responses for positive stimuli.

**AGN Total omissions (negative):** Number of missed responses for negative stimuli.

**AGN Total omissions (neutral):** Number of missed responses for neutral stimuli.

**eTable 1: FT Study, fROI Brain Analysis**

| **Time-Point** | **Contrast** | **Analysis** | **MNI coordinates** | | | **Anatomical Area** | **K** | **p(FWE-corr)**  **Cluster Level** | **p(FWE-corr)**  **Peak Level** | **T score** | **Z score** |
| --- | --- | --- | --- | --- | --- | --- | --- | --- | --- | --- | --- |
|  |  |  | **X** | **Y** | **Z** |  |  |  |  |  |  |
| BL | Angry-Control | High>Low | 42 | 8 | -14 | Right Insular Cortex, BA13 | 2 | 0.007 | 0.016* | 5.19 | 5.06 |
| BL | Angry-Neutral | Group Average Positive | -36 | 11 | -5 | Left Insular Cortex, BA13 | 1 | NaN | 0.001 | 5.67 | 5.51 |
| FU | Angry-Control | Group Average Positive | -12 | 5 | 10 | Left Caudate Body | 7 | <0.0001 | 0.001 | 5.73 | 5.56 |
| FU | Angry-Control | Group Average Positive | 6 | 65 | 31 | Right Frontal Lobe, Superior Frontal Gyrus, BA10 | 99 | <0.0001 | <0.0001 | 7.82 | 7.39 |
| FU | Angry-Control | Group Average Negative | 33 | -46 | -5 | Right Limbic Lobe, Parahippocampal Gyrus, BA19 | 12543 | NaN | <0.0001 | 16.77 | Inf |
| **Abbreviations**  **BL:** Baseline, age 14; **FU:** Follow-up, age 19; **High Group**: Scorers in upper 10% of CAPE Total Score, n=149; **Low Group:** Scorers in lower 10% CAPE Total Score, n=149; **High>Low:** showing increased activation in the High but not in the Low Group; **Low>High:** showing increased activation in the Low but not in the High Group; **Group Average Positive:** showing increased activation in both High and Low Groups; **Group Average Negative:** showing decreased activation in both High and Low Groups; **BA:** Brodmann Area; **K:** number of Voxels; **p(FWE-corr):** p value corrected for Family Wise Error (false positives); **NaN:** Not a Number; **Inf:** Infimum; **(*):** did not survive Bonferoni correction for multiple testing at p=0.01 (0.05/5). | | | | | | | | | | | |

**eTable 2: FT Study, Exploratory Cross-Sectional Analysis, Independent T-tests.**

| **Time-Point** | **MNI Coordinates** | | | **Anatomical Area** | **High Group Mean Brain Activation (Parameter Estimates)** | **Low Group Mean Brain Activation (Parameter Estimates)** | **Group Activation Relation** | **t-test for Equality of Means** | | | |
| --- | --- | --- | --- | --- | --- | --- | --- | --- | --- | --- | --- |
|  | **X** | **Y** | **Z** |  |  |  |  | **t-test** | **df** | **Sig.** | **r** |
| BL | 42 | 8 | -14 | Right Insular Cortex, BA13 | 0.146 | -0.269 | H>L | 5.188 | 264 | <0.0001 | 0.304 |
| BL | -36 | 11 | -5 | Left Insular Cortex, BA13 | 0.094 | 0.203 | L>H | -2.063 | 262 | 0.04 | 0.126 |
| FU | -12 | 5 | 10 | Left Caudate Body | 0.066 | 0.173 | L>H | -2.588 | 255 | 0.01 | 0.160 |
| FU | 6 | 65 | 31 | Right Frontal Lobe, Superior Frontal Gyrus, BA10 | 0.245 | 0.446 | L>H | -2.283 | 257 | 0.023 | 0.141 |
| FU | 33 | -46 | -5 | Right Limbic Lobe, Parahippocampal Gyrus, BA19 | -0.458 | -0.324 | L>H | -2.85 | 255 | 0.005 | 0.176 |
| **Abbreviations**  **BL:** Baseline, age 14; **FU:** Follow-up, age 19; **BA:** Brodmann Area; **H>L:** High Group showing more activation than Low Group; **L>H:** Low Group showing more activation than High Group; **r:** Pearson’s correlation coefficient; **df:** degrees of Freedom | | | | | | | | | | | |

**eTable 3: FT Study, Exploratory Longitudinal Analysis, Paired T-tests.**

| **MNI Coordinates** | | | **Anatomical Area** | **Groups** | **Baseline Mean Brain Activation (Parameter Estimates)** | **Follow-up Mean Brain Activation (Parameter Estimates)** | **Group Activation Relation** | **t-test for Equality of Means** | | | |
| --- | --- | --- | --- | --- | --- | --- | --- | --- | --- | --- | --- |
| **X** | **Y** | **Z** |  |  |  |  |  | **t-test** | **df** | **Sig.** | **r** |
| 33 | -46 | -5 | Right Limbic Lobe, Parahippocampal Gyrus, BA19 | HIGH & LOW | -0.267 | -0.384 | BL>FU | 3.481 | 231 | 0.001 | 0.223 |
|  |  |  |  | HIGH | -0.283 | -0.456 | BL>FU | 4.307 | 116 | <0.0001 | 0.371 |
|  |  |  |  | LOW | -0.250 | -0.311 | BL>FU | 1.129 | 114 | 0.261* | 0.105 |
| 6 | 65 | 31 | Right Frontal Lobe, Superior Frontal Gyrus, BA10 | HIGH & LOW | 0.224 | 0.367 | FU>BL | -2.614 | 231 | 0.01 | 0.169 |
|  |  |  |  | HIGH | 0.203 | 0.250 | FU>BL | -0.579 | 116 | 0.564* | 0.054 |
|  |  |  |  | LOW | 0.246 | 0.487 | FU>BL | -3.311 | 114 | 0.001 | 0.296 |
| 42 | 8 | -14 | Right Insular Cortex, BA13 | HIGH & LOW | -0.065 | -0.064 | FU>BL | -0.017 | 231 | 0.987* | 0.001 |
|  |  |  |  | HIGH | 0.157 | -0.028 | BL>FU | 1.828 | 116 | 0.07* | 0.167 |
|  |  |  |  | LOW | -0.291 | -0.1 | FU>BL | -2.05 | 114 | 0.043 | 0.188 |
| -36 | 11 | -5 | Left Insular Cortex, BA13 | HIGH & LOW | 0.151 | -0.198 | BL>FU | 8.043 | 228 | <0.0001 | 0.470 |
|  |  |  |  | HIGH | 0.081 | -0.225 | BL>FU | 4.984 | 114 | <0.0001 | 0.423 |
|  |  |  |  | LOW | 0.222 | -0.170 | BL>FU | 6.399 | 113 | <0.0001 | 0.516 |
| **Abbreviations**  **BA:** Brodmann Area; **BL>FU:** Baseline activation greater than Follow-up activation; **FU>BL:** Follow-up activation greater than Baseline activation; **r:** Pearson’s correlation coefficient, **(*)**: not statistically significant at a p=0.05 level; **df:** degrees of Freedom.  **High**: Scorers in upper 10% of CAPE Total Score, n=149; **Low:** Scorers in lower 10% CAPE Total Score, n=149. | | | | | | | | | | | |

**eTable 4: CANTAB Measures Exploratory Cross-Sectional Analysis,** **Independent T-tests.**

| **CANTAB Variable** | **Timepoint** | **High Group Mean Score** | **Low Group Mean Score** | **Group Scores Relation** | **t-test for Equality of Means** | | | |
| --- | --- | --- | --- | --- | --- | --- | --- | --- |
|  |  |  |  |  | **t-test** | **df** | **Sig.** | **r** |
| AGN Total Omissions Negative | BL | 10.052 | 13.177 | L>H | -3.021 | 235 | 0.003 | 0.193 |
| AGN Total Omissions Positive | BL | 12.035 | 14.645 | L>H | -2.817 | 237 | 0.005 | 0.180 |
| **Abbreviations**  **AGN Total Omissions Negative/Positive:** Affective Go-NoGo Task, total number of missed responses to targets in the blocks specified by the value of target type (negative, positive); **CGT Risk Adjustment:** Cambridge Gambling Task, tendency to get higher proportions of points when the large majority of boxed are the colour chosen; **L>H:** Low Group showing greater scores than High Group; **r:** Pearson’s correlation coefficient; **BL:** Baseline, age 14; **df:** degrees of Freedom. | | | | | | | | |

**eTable 5: CANTAB Measures Exploratory Longitudinal Analysis, Paired T-tests.**

| **CANTAB Variable** | **Group** | **Baseline Mean Score** | **Follow-up Mean Score** | **Group Scores Relation** | **t-test for Equality of Means** | | | |
| --- | --- | --- | --- | --- | --- | --- | --- | --- |
|  |  |  |  |  | **t-test** | **df** | **Sig.** | **r** |
| AGN Total Omissions Negative | ALL | 11.899 | 6.647 | BL>FU | 18.932 | 781 | <0.0001 | 0.561 |
|  | HIGH | 10.065 | 6.688 | BL>FU | 3.745 | 76 | <0.0001 | 0.395 |
|  | LOW | 12.369 | 7.095 | BL>FU | 6.385 | 83 | <0.0001 | 0.574 |
| AGN Total Omissions Positive | ALL | 13.787 | 8.551 | BL>FU | 19.853 | 781 | <0.0001 | 0.579 |
|  | HIGH | 12.195 | 8.273 | BL>FU | 4.529 | 76 | <0.0001 | 0.461 |
|  | LOW | 14.274 | 8.964 | BL>FU | 6.352 | 83 | <0.0001 | 0.572 |
| **Abbreviations**  **AGN Total Omissions Negative/Positive:** Affective Go-NoGo Task, total number of missed responses to targets in the blocks specified by the value of target type (negative, positive); **BL>FU:** Mean Scores greater at BL; **r:** Pearson’s correlation coefficient; **BL:** Baseline, age 14; **FU:** Follow-up, age 19: **ALL:** Whole sample, n=1,434; **df:** degrees of Freedom. | | | | | | | | |

**eTable 6: CAPE-42 Clusters**

| **1 Depressive Symptoms** | | **2 Positive Symptoms** | | | | **3 Negative Symptoms** | |
| --- | --- | --- | --- | --- | --- | --- | --- |
|  |  | **2a** | **Bizarre Symptoms** | **2b** | **Social Delusions** |  |  |
| **Q1** | Sad | **Q5** | Messages from Media | **Q2** | Double Meaning | **Q3** | Not animated |
| **Q9** | Pessimism | **Q15** | Telepathy | **Q6** | False Appearance | **Q4** | Not talkative |
| **Q12** | No future | **Q17** | Influenced by devices | **Q7** | Being persecuted | **Q8** | No emotions |
| **Q14** | Not worth living | **Q20** | Voodoo | **Q10** | Conspiracy | **Q16** | No interest in others |
| **Q19** | Frequently Crying | **Q22** | Odd Looks | **Q11** | Being important | **Q18** | Lack of motivation |
| **Q38** | Guilty | **Q24** | Thought Withdrawal | **Q13** | Being special | **Q21** | No energy |
| **Q39** | Feeling a failure | **Q26** | Thought Insertion |  |  | **Q23** | Empty mind |
| **Q40** | Feeling tense | **Q28** | Thought Broadcasting |  |  | **Q25** | Lack of activity |
|  |  | **Q30** | Thought Echo |  |  | **Q27** | Lack of emotional intensity |
|  |  | **Q31** | External Control |  |  | **Q29** | Lack of spontaneity |
|  |  | **Q33** | Auditory Hallucinations |  |  | **Q32** | Blunted emotions |
|  |  | **Q34** | Voices Conversing |  |  | **Q35** | Lack of hygiene |
|  |  | **Q41** | Capgras |  |  | **Q36** | Unable to finish |
|  |  | **Q42** | Visual Hallucinations |  |  | **Q37** | Lack of interests |

**eTable 7: Correlations between selected CAPE Scores, overall sample (n=1,434)**

|  | CAPE Grand Total | CAPE Positive Symptoms Total | CAPE Bizarre Delusions Total | CAPE Social Delusions Total | CAPE Negative Symptoms Total |
| --- | --- | --- | --- | --- | --- |
| CAPE Grand Total | 1.000 | **0.790**  (p<0.0001) | **0.558**  (p<0.0001) | **0.761**  (p<0.0001) | **0.913**  (p<0.0001) |
| CAPE Positive Symptoms Total | **0.790**  (p<0.0001) | 1.000 | **0.755**  (p<0.0001) | **0.931**  (p<0.0001) | **0.584**  (p<0.0001) |
| CAPE Bizarre Delusions Total | **0.558**  (p<0.0001) | **0.755**  (p<0.0001) | 1.000 | **0.497**  (p<0.0001) | **0.405**  (p<0.0001) |
| CAPE Social Delusions Total | **0.761**  (p<0.0001) | **0.931**  (p<0.0001) | **0.497**  (p<0.0001) | 1.000 | **0.567**  (p<0.0001) |
| CAPE Negative Symptoms Total | **0.913**  (p<0.0001) | **0.584**  (p<0.0001) | **0.405**  (p<0.0001) | **0.567**  (p<0.0001) | 1.000 |
| **In bold:** Spearman’s rho (correlation coefficient), non-parametric  **p:** 2-tailed significance level | | | | | |

**eFigure 1: CAPE Total Scores Frequencies Histogram, overall sample (n=1,434)**

**eFigure 2: CANTAB Measures, Mean AGN Total Omissions Positive and Negative Stimuli Scores at age 14 and 19;** statistically significant changes at p=0.05 level for both the High and Low PLEs Group, SE bars are displayed
